# Enhanced Biphenyl Degradation by *Rhodococcus* sp. TG-1 Under Cr(VI) Stress via Modified Biochar Immobilization

**DOI:** 10.3390/microorganisms14061384

**Published:** 2026-06-22

**Authors:** Ying Zhai, Lei Huang, Xiuwei Hou, Yuefeng Zou, Xin Zhao, Meitong Li

**Affiliations:** 1 Tianjin Key Laboratory of Organic Solar Cells and Photochemical Conversion, College of Chemistry and Chemical Engineering, Tianjin University of Technology, Tianjin 300384, China; xiaozhai@stud.tjut.edu.cn (Y.Z.); huanglei@tjut.edu.cn (L.H.); 20240957@stud.tjut.edu.cn (X.H.); zouyuefeng1@stud.tjut.edu.cn (Y.Z.); 2 School of Resources and Civil Engineering, Northeastern University, Shenyang 110819, China

**Keywords:** biphenyl, biodegradation, Cr(VI), microbial immobilization

## Abstract

Co-contamination of biphenyl and heavy metals is widespread in industrial environments, but systematic studies on the simultaneous treatment of both pollutants using a single microbial strategy remain limited. In this study, we characterized the biphenyl degradation performance, metabolic pathway, transcriptomic response, and Cr(VI) tolerance of *Rhodococcus* sp. TG-1, and developed an alkali-modified biochar immobilization system to enhance its degradation efficiency for biphenyl under Cr(VI) stress. Degradation experiments were carried out under optimal conditions (30 °C, pH 7.0), and it was found that strain TG-1 degraded 76.84% of 300 mg/L biphenyl within 3 days. Intermediate metabolites were identified by LC-MS, and five key intermediates were detected, confirming that TG-1 metabolizes biphenyl via the classical 2,3-dihydroxybiphenyl dioxygenase pathway, with subsequent entry into the tricarboxylic acid cycle. Transcriptomic analysis was performed to profile gene expression, revealing 845 differentially expressed genes under biphenyl stress, including 672 upregulated genes significantly enriched in aromatic degradation pathways. Seven complete *bph* gene clusters responsible for biphenyl catabolism were also identified. Strain TG-1 exhibited high tolerance to Cr(VI), with a minimum inhibitory concentration (MIC) of 500 mg/L. However, its biphenyl degradation efficiency dropped to 51.32% in the presence of 200 mg/L Cr(VI). After immobilization using alkali-modified straw biochar (JBC), heavy metal toxicity was alleviated, and the biphenyl removal rate increased to 99.30% under co-contamination conditions. Scanning electron microscopy (SEM) and Fourier transform infrared spectroscopy (FTIR) analyses confirmed that TG-1 was stably loaded onto the biochar surface through hydrogen bonding and electrostatic interactions. Altogether, this study provides a promising bacterial strain and a green immobilization strategy for enhancing biphenyl removal in the presence of Cr(VI), offering a practical approach for the treatment of environments co-contaminated with aromatic compounds and heavy metals.

## 1. Introduction

Biphenyl is a typical refractory aromatic pollutant widely distributed in industrial environments, which is inevitably produced as a byproduct during coal refining, petroleum processing, and natural gas exploitation [1]. Incomplete combustion of coal, petroleum, and fossil fuels has been demonstrated to result in substantial releases of biphenyl into soil and aquatic environments [2,3]. Long-term exposure to biphenyl has been confirmed to induce multiple toxic effects in humans, including muscle atrophy, ophthalmic irritation, renal dysfunction, bladder cancer, and damage to the central nervous system and liver [4,5].

Biphenyl is constituted by two benzene rings which are linked by a stable π-electron system. This endows the compound with extremely high structural stability and resistance to natural degradation [6]. In the context of biphenyl-contaminated soil and water bodies, conventional remediation strategies predominantly employ physical and chemical methodologies. These methodologies encompass high-temperature incineration and alkali-catalysed decomposition [4,6,7]. Nonetheless, the deployment of such technologies is often associated with significant financial investment, intricate operational processes, substantial energy consumption, and the challenge of achieving complete mineralisation. This has the potential to result in secondary pollution [8,9]. In comparison, microbial remediation can be regarded as an eco-friendly, cost-effective, and sustainable alternative, owing to its high mineralisation efficiency and environmental compatibility [10]. As demonstrated in earlier studies, microbial oxygenases and oxidoreductases facilitate the efficient catabolism of biphenyl, exhibiting a broad substrate spectrum. This supports the degradation of various aromatic pollutants, including biphenyl, polychlorinated biphenyls, polycyclic aromatic hydrocarbons, toluene, and chlorobenzene [11,12].

In industrial zones, coking wastewater systems, and coal-related contaminated sites, biphenyl commonly coexists with highly toxic heavy metals; among them, Cr(VI) is of particular concern due to its strong oxidizing capacity, high solubility, and pronounced synergistic toxicity when combined with aromatic hydrocarbons [13,14,15]. Such combined pollution triggers synergistic toxic effects, inhibiting microbial activity and disrupting intracellular metabolic pathways. In addition, these effects have been observed to intensify competitive adsorption and reduce pollutant bioavailability, thereby significantly increasing the difficulty of remediation [16,17,18]. Conventional physical and chemical technologies have proven ineffective in simultaneously and efficiently removing organic pollutants and heavy metals. In contrast, free microbial remediation is severely restricted by heavy metal toxicity [19]. In recent years, biochar-immobilized microbial technology has emerged as a promising strategy for combined pollution remediation [20,21]. It has been demonstrated that biochar has the capacity to provide a stable microhabitat for functional microorganisms, to adsorb heavy metals in order to alleviate their toxicity, and to enhance the degradation efficiency of refractory organic pollutants [21,22]. Consequently, the construction of an immobilized microbial system that possesses both heavy metal tolerance and superior biphenyl degradation capacity is of critical importance for the green and efficient remediation of biphenyl-contaminated environments. In this study, 200 mg/L Cr(VI) was selected because this concentration falls within the typical range of Cr(VI)-contaminated industrial wastewater (80–250 mg/L) [13], and simultaneously represents a sub-inhibitory level (below the MIC of 500 mg/L), allowing us to assess both stress responses and practical remediation potential.

Biochar is a carbon-rich porous material derived from plant biomass or organic waste via high-temperature pyrolysis under oxygen-limited conditions [23]. Numerous studies have validated that biochar possesses excellent adsorption capacity toward various pollutants and can serve as an ideal carrier for microbial immobilization, thereby improving microbial survival, colonization, and catalytic performance [24,25]. Microbial immobilization refers to the attachment of functional microorganisms onto suitable carriers via physical or chemical interactions, which facilitates microbial proliferation, activity retention, and metabolic stability [26]. Although raw biochar can promote pollutant degradation, its practical application is often limited by relatively low porosity, insufficient surface functional groups, and inadequate nutrient supply compared with activated carbon [27]. Accordingly, various modification strategies have been developed to optimize the structural and surface properties of biochar, mainly including physical modification, chemical modification, and nanomaterial-assisted modification, among which chemical modification is the most widely applied [28]. Chemical modification significantly affects surface hydrophilicity, enriching oxygen-containing functional groups [24]. Specifically, acid modification introduces acidic groups and strengthens metal adsorption affinity, while alkali modification generates abundant oxygen-containing groups, reduces ash content, and regulates surface charge characteristics [24].

*Rhodococcus* sp. TG-1 was isolated from petroleum-contaminated soil. Much of the earlier work focused only on degradation efficiency or single-factor optimization. In contrast, this strain can utilize crude oil and toluene as sole carbon sources and maintain high degradation activity under high salinity stress [29], indicating that TG-1 harbors a complete aromatic hydrocarbon catabolic pathway and strong environmental adaptability. However, its biphenyl degradation pathway, transcriptomic response, and co-tolerance to Cr(VI) during immobilization have not been systematically explored. Accordingly, this study integrates pathway elucidation via LC-MS, transcriptomic analysis under biphenyl stress, Cr(VI) tolerance evaluation, and alkali-modified biochar immobilization into a single investigation. An immobilized microbial system was further constructed using alkali-modified straw biochar to enhance stress resistance and improve biphenyl removal in the presence of Cr(VI) co-contamination. This work provides a high-efficiency bacterial strain and a green, feasible strategy for the removal of aromatic compounds from co-contaminated sites.

## 2. Materials and Methods

### 2.1. Bacteria and Culture Media

Minimal salt medium (MSM) and Luria–Bertani (LB) medium were prepared according to previous studies [30,31]. Biphenyl (≥99% purity) was obtained from Shanghai Macklin Biochemical Technology Co., Ltd. (Shanghai, China). Dichloromethane (98% purity) was supplied by Tianjin Standard Chemical Reagents Co., Ltd. (Tianjin, China). Anhydrous ethanol (≥99.7% purity) was purchased from Tianjin Damao Chemical Reagents Partnership (Tianjin, China). Straw biochar was purchased from Pingdingshan Tanerno New Materials Co., Ltd. (Pingdingshan, Henan, China). All other chemical reagents were purchased from Tianjin Damao Chemical Reagents Factory (Tianjin, China).

This *Rhodococcus* strain TG-1 was isolated from oil-contaminated soil collected from the Liaohe Oilfield in China (41.13–41.26° N, 121.08–121.09° E). The raw genomic data of this strain has been deposited in the NCBI database and is available under the BioProject accession PRJNA739011 in the Sequence Read Archive (SRA), with the BioSample accession SAMN19769069 and GenBank accession numbers CP077417, CP077418, CP077419, and CP077420 [29]. Through preliminary experiments, it was found that this strain exhibited good biphenyl degradation performance. When *Rhodococcus* sp. TG-1 was inoculated into the inorganic salt medium containing only biphenyl as the sole carbon source, the medium turned yellow on the 2nd day of cultivation. With the extension of cultivation time, the yellow color first deepened and then faded until it completely disappeared, indicating that the strain could effectively metabolize biphenyl, which is the key reason for selecting this strain for subsequent biphenyl degradation experiments.

Seed medium and degradation medium (g/L): (NH_4_)_2_SO_4_ 4 g, Na_2_HPO_4_·12H_2_O 12.5 g, KH_2_PO_4_ 2.04 g, MgSO_4_ 0.1 g, yeast powder 0.01 g, pH 7.2, autoclaved at 121 °C for 30 min; add 2% anhydrous ethanol upon inoculation to prepare the seed medium. To avoid carbon source carryover, the seed culture was centrifuged (6000 rpm, 10 min) and washed twice with sterile MSM before inoculation. The resulting cell pellet was resuspended in MSM prior to use. Biphenyl was prepared as a stock solution in dichloromethane, sterilized by filtration through a 0.22 μm organic filter membrane, and added to the sterilized inorganic salt medium. The solvent (dichloromethane) was then evaporated under aseptic conditions before inoculation to eliminate any potential carbon contribution.

LMM medium (g/L): KH_2_PO_4_ 0.1, Na_2_HPO_4_ 0.1, NH_4_NO_3_ 0.5, (NH_4_)_2_SO_4_ 0.5, MgSO_4_ 0.2, CaCl_2_ 0.02, FeCl_2_ 0.002, and MnSO_4_ 0.002; pH 6.5.

### 2.2. Determination of Biphenyl Degradation Rate

The *Rhodococcus* sp. TG-1 strain was cultivated in minimal medium (MM) with biphenyl as the sole carbon source. Biphenyl degradation experiments were carried out at initial concentrations of 100, 300, 500, 700, and 900 mg/L. A 2% inoculum of TG-1 was added to each medium, and the cultures were incubated at 30 °C on a shaking incubator at 150 rpm for 3 days. Cell growth was monitored by measuring the absorbance at 600 nm (Abs). The residual biphenyl concentration was determined by gas chromatography, and the degradation rate was then calculated.

For sample preparation, an equal volume of ethyl acetate was added to the culture medium and extracted for 20 min. The supernatant was collected, and the extraction was repeated three times. The combined organic phases were dried over anhydrous sodium sulfate, filtered, and mixed with phenanthrene (30 g/L) as an internal standard before GC analysis. Biphenyl quantification was performed using a gas chromatograph (Agilent 7890A) equipped with a flame ionization detector (FID), manufactured by Agilent Technologies, Santa Clara, CA, USA. A 10-μL aliquot of the organic phase was injected into an Agilent HP-5 capillary column (30 m × 0.32 mm I.D., 0.25 μm film thickness). The oven temperature was programmed as follows: hold at 80 °C for 1 min, ramp to 280 °C at 10 °C/min, and hold at 280 °C for 10 min.

The biphenyl degradation rate was calculated using the following equation:

Degradation rate (%) = [(C_0_ − C_t_)/C_0_] × 100%

where C_0_ is the initial biphenyl concentration (mg/L), and C_t_ is the residual biphenyl concentration (mg/L) after incubation. To ensure that the observed biphenyl removal was indeed due to microbial degradation rather than abiotic factors or solvent effects, two types of controls were included in all experiments: (i) a solvent control (dichloromethane without biphenyl, processed identically) to confirm that the solvent itself does not support bacterial growth or serve as a carbon source and (ii) an abiotic control (biphenyl-containing medium without inoculation) to account for any biphenyl loss caused by adsorption, volatilization, or photolysis under the same experimental conditions.

### 2.3. Effect of Different Cultivation Conditions on the Degradation Rate of Biphenyl

A 300 mg/L solution of biphenyl was used for subsequent experiments; the cultivation methods and the method for determining biphenyl concentration were the same as those described in Section 2.2. A single-factor optimization experiment was designed, varying the following parameters: days (1, 3, 5, 7), pH (5, 6, 7, 8, 9), and temperature (20, 25, 30, 35 °C). For the experiments investigating pH and temperature, all culture media were incubated for 3 days, which was the optimal cultivation time determined from the previous experiment. For the experiment investigating the effect of cultivation days, the incubation time was set as 1, 3, 5, and 7 days, respectively. The residual biphenyl concentration in the culture media was measured, and the biphenyl degradation rate was calculated.

### 2.4. Identification of Biphenyl Degradation Intermediates

Following a period of three days during which the system was subjected to incubation under conditions that could be described as optimal, the degradation system was subjected to three liquid–liquid extractions using an equal volume of ethyl acetate. The organic phases were then amalgamated, desiccated over anhydrous sodium sulfate, and filtered. The mixture was then subjected to vacuum distillation in a 35 °C water bath, resulting in the concentration of the substance. The residue was dissolved in chromatographic-grade methanol, filtered through a 0.45 μm organic filter membrane, and analysed by liquid chromatography–mass spectrometry (LC-MS).

LC-MS analysis was performed on a Waters HPLC-MS system. Metabolites were separated on a Shimadzu ODS-3 column (5 μm, 4.6 mm × 250.0 mm; MetaChem Technologies) equipped with a guard column packed with the same stationary phase, connected to a Waters 515 solvent delivery system. The mobile phase consisted of a programmed methanol/water gradient at a flow rate of 1.0 mL/min. The gradient program was as follows: linear gradient from 50% to 95% (*v*/*v*) methanol over 45 min, held at 95% methanol for 10 min, then returned from 95% to 50% methanol over 5 min. Electrospray ionization (ESI) was employed, and data were acquired in negative ion mode (both positive and negative modes were tested). The drying gas (N_2_) flow rate was 15 L/min, and the mass scan range was set from 50 to 500 *m*/*z*.

### 2.5. Gene Expression Analysis

#### 2.5.1. Transcriptome Analysis

To investigate the molecular response mechanisms of *Rhodococcus* sp. under exposure to 300 mg/L biphenyl, this study utilized transcriptomic sequencing to analyze gene expression differences. Biphenyl was dissolved in dichlorotoluene. Strain TG-1 was inoculated at a 2% concentration into a basal inorganic salt medium containing 300 mg/L biphenyl. A control group was established by inoculating strain TG-1 at a 2% concentration into a basal inorganic salt medium without biphenyl. Three independent biological replicates were prepared for each group. After 72 h of incubation, the cells were harvested by centrifugation at 10,000 rpm for 10 min at 4 °C. The cells were washed three times with pre-chilled PBS, the supernatant was discarded, and the cells were frozen in liquid nitrogen for 10 min. The frozen samples were stored at −80 °C for subsequent RNA extraction. Transcriptome sequencing was performed by Meiji Biotechnology (Shanghai, China) on the NovaSeq X Plus platform (Illumina, San Diego, CA, USA).

Total RNA was extracted using the TiGen RNAprep Kit (TiGen, Beijing, China) according to the manufacturer’s instructions, and genomic DNA was removed using DNase I (Takara, Kusatsu, Japan). RNA integrity was assessed via agarose gel electrophoresis, and the concentration and purity of the extracted RNA were measured using a Nanodrop 2000 (Thermo Fisher Scientific, Waltham, MA, USA). The RNA integrity number (RIN value) was determined using an Agilent 2100 Bioanalyzer (Agilent Technologies, Santa Clara, CA, USA).

Ribosomal RNA was removed using a Ribo-Zero™ Plus kit (Thermo Fisher Scientific). Libraries were prepared with a TruSeq™ Stranded Total RNA Library Prep Kit (dUTP/UNG method). PE150 sequencing generated approximately 12.5 million clean reads per sample (fastp filtering, Q < 20). Clean reads were aligned to the reference genome of *Rhodococcus* sp. TG-1 (NCBI PRJNA739011) using Bowtie2. Gene expression was quantified by RSEM (TPM). Differential expression analysis was performed using DESeq2, with |log_2_FC| ≥ 1 and FDR < 0.05 as the significance threshold.

#### 2.5.2. Real-Time Quantitative Fluorescence PCR (qRT-PCR) Validation Analysis

qRT-PCR assays were run on a Q9600 series real-time fluorescence quantitative PCR instrument (Bio-Gener Technology Co., Hangzhou, China). The 2× RealStar Fast dye-based qPCR premix (GenStar, Beijing, China) was used as follows: 10 µL of 2× RealStar Fast SYBR qPCR premix, 0.5 µL of forward primer (10 µM), 0.5 µL of reverse primer (10 µM), 0.4 µL of High/Low ROX Reference Dye, and 1 µL of DNA template. The optimal cycling conditions were: pre denaturation at 95 °C for 2 min, followed by 40 cycles of amplification, with each cycle consisting of denaturation at 95 °C for 15 s, annealing at 54 °C for 20 s, and extension at 72 °C for 30 s; fluorescence signals were collected during the extension stage throughout the entire process. The 2^−ΔΔCt^ method was used to calculate the relative expression levels of the target genes. All samples were run independently three times.

### 2.6. Testing of Strain Tolerance to Heavy Metal Cr(VI)

A stock solution of metal ions with a concentration of 5000 mg/L was prepared using K_2_Cr_2_O_7_ (sterilized by filtration through a 0.45 μm filter membrane). Cr(VI) was added to 50 mL LMM medium containing 300 mg/L biphenyl, followed by inoculation with 2% (*v*/*v*) seed culture of *Rhodococcus* sp. TG-1. After incubation at 30 °C with shaking at 150 rpm for 3 days, the minimum inhibitory concentration (MIC) of Cr(VI) toward the strain was determined. A control group was established using medium inoculated with only the TG-1 strain to assess the strain’s abiotic loss. Five concentrations (50, 100, 200, 300, and 500 mg/L) were selected within the MIC range to test the degradation rate of biphenyl. The hexavalent chromium concentration most closely resembling actual contamination scenarios was selected as the experimental concentration for the subsequent dual-pollutant degradation system.

### 2.7. Preparation of Alkali-Modified Biochar and Its Immobilized Microbial Inoculants

Straw biochar was selected for modification, and subjected to alkali treatment with 1 mol/L NaOH solution according to the method described by Mahdi [31]. Briefly, 10 g of straw biochar was added to 50 mL of 1 mol/L NaOH solution at a solid-to-liquid ratio (*w*:*v*) of 1:5, followed by shaking immersion for 4 h. The mixture was then rinsed with deionized water until the supernatant reached a neutral pH, and subsequently filtered under vacuum. The resulting solid was dried in an oven at 100 °C for 2 h to obtain the alkali-modified biochar, designated as JBC.

Straw biochar (BC) and alkali-modified straw biochar (JBC) were sieved through 50-mesh and 100-mesh standard sieves, accurately weighed, and transferred into 100 mL conical flasks with 40 mL of mineral salt medium. After autoclaving, 2% (*v*/*v*) of *Rhodococcus* sp. TG-1 seed culture was inoculated, followed by shaking incubation at 30 °C and 150 rpm for 12 h to achieve adsorption and immobilization of the strain on the biochar surface. The immobilized products were designated as TBC (BC loaded with TG-1) and JTBC (JBC loaded with TG-1).

### 2.8. Degradation of Cr(VI)-Biphenyl Co-Pollutants by Immobilized Bacteria

In previous laboratory studies, straw biochar was subjected to acid and alkali modification, and its characterization (SEM, FTIR, XPS, BET) was analyzed before and after modification. The results showed that the performance of alkali-modified straw biochar (BC-BW) was significantly improved [31,32]. Therefore, in subsequent experimental studies on the degradation of dual contamination by heavy metals and biphenyl, alkali-modified biochar was selected as the experimental material. Optimization experiments were first conducted to determine the optimal dosage of alkali-modified biochar. Subsequently, alkali-modified straw biochar (at the optimal dosage) was combined with strain TG-1 to prepare a fixed bacterial agent, using the same method described in Section 2.7. This immobilized bacterial preparation was added to LMM liquid medium containing 300 mg/L of biphenyl and 200 mg/L of Cr(VI), and the mixture was incubated on a shaking incubator at 30 °C and 150 rpm for 3, 5, 7, and 9 days. The residual biphenyl concentration was determined according to the method described in Section 2.2, and the degradation rate was calculated.

### 2.9. Data Analysis

All experiments described above were conducted with three biological replicates, and data are presented as mean ± standard deviation (SD). Data analysis, statistical processing, and graphing were performed using Origin 2025, IBM SPSS Statistics 27, and GraphPad Prism 9.5 software. One-way analysis of variance (ANOVA) was used to assess the significance of differences between groups, with *p* < 0.05 serving as the threshold for statistical significance.

## 3. Results and Discussion

### 3.1. Single-Factor Optimization Experiment for Biphenyl Degradation

**Figure 1:**
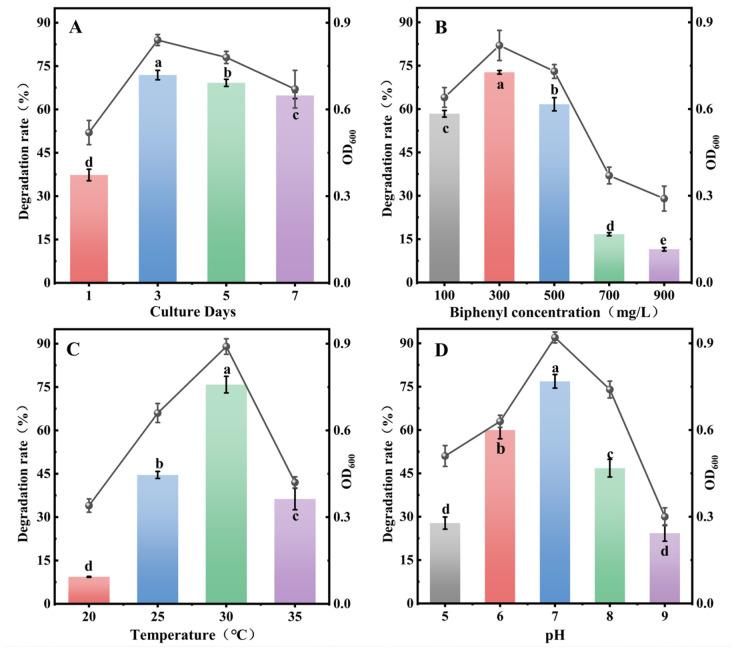
Optimization of culture conditions for biphenyl degradation by *Rhodococcus* sp. TG-1. (**A**) Culture time, (**B**) biphenyl concentration, (**C**) temperature, (**D**) pH. Error bars show standard deviations of three replicates. Colored bars stand for biphenyl degradation rate (%, left y-axis), and lines with circle markers show cell growth (OD_600_, right y-axis). Different lowercase letters above bars mean significant intergroup differences by one-way ANOVA and Duncan’s multiple range test (*p* < 0.05). Error bars denote SD of three independent biological replicates.

To determine the optimal conditions for biphenyl degradation by *Rhodococcus* sp. TG-1, the effects of incubation time, initial substrate concentration, temperature, and pH were systematically evaluated via single-factor experiments. The time-dependent degradation profile was first investigated at 25 °C, 150 rpm, pH 7.0, and 300 mg/L biphenyl. As depicted in Figure 1A, the degradation efficiency increased rapidly within the initial 3 days and reached a maximum of 71.88% at day 3. The subsequent slight decrease could be attributed to nutrient exhaustion, the entry of cells into the decline phase, and the accumulation of toxic metabolic intermediates, which collectively suppressed the catalytic activity of strain TG-1 [33,34,35]. Thus, 3 days was determined as the optimal incubation duration for all subsequent experiments.

The effect of initial biphenyl concentration was further optimized (Figure 1B). Strain TG-1 exhibited efficient degradation capacity over a wide concentration range of 100–500 mg/L, with all degradation ratios exceeding 50% within 3 days, indicating its strong environmental adaptability. The highest degradation efficiency of 72.71% was achieved at 300 mg/L, which was therefore selected as the optimal substrate concentration. As illustrated in Figure 1C, the degradation efficiency peaked at 75.82% at 30 °C, while lower (20 °C) or higher (35 °C) temperatures significantly inhibited degradation activity due to the suppressed catalytic efficiency of functional enzymes [36]. Similarly, as shown in Figure 1D, degradation was inefficient under strongly acidic (pH 5) or alkaline (pH 9) conditions, and the maximum efficiency of 76.84% was obtained at pH 7.0. Extreme pH values disrupt cell membrane stability and intracellular metabolic homeostasis, thereby inhibiting biphenyl catabolism [37,38]. Accordingly, pH 7.0 was determined as the optimal pH.

Collectively, the optimal conditions for biphenyl degradation by *Rhodococcus* sp. TG-1 were determined as 30 °C, pH 7.0, 300 mg/L initial biphenyl concentration, and 3 days of incubation.

### 3.2. Identification of Biphenyl Degradation Intermediates and Inference of Metabolic Pathways

To elucidate the catabolic mechanism of biphenyl by strain TG-1, key intermediates were identified using LC-MS (Figure S1). Five critical metabolites were unambiguously detected: Biphenyl, 2,3-dihydroxybiphenyl, 2-hydroxy-6-oxo-6-phenylhexa-2,4-dienoic acid (HOPDA), 2-hydroxypenta-2,4-dienoic acid, and 2-oxoglutaric acid (α-ketoglutaric acid). Microbial biphenyl degradation typically follows the classical 2,3-dihydroxybiphenyl dioxygenase pathway [39]. Based on the identified metabolites, we reconstructed the complete metabolic pathway, which is highly consistent with the classical route reported in previous studies [39,40].

**Figure 2:**
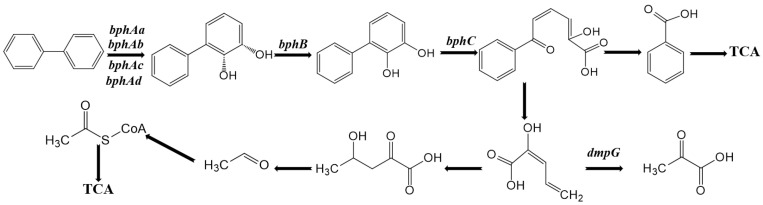
Metabolic pathway of *Rhodococcus* sp. TG-1 for the degradation of biphenyl. Genes labeled on arrows encode corresponding catabolic enzymes: *bphAa/bphAb* (large/small subunits of biphenyl 2,3-dioxygenase), *bphAc* (ferredoxin component), *bphAd* (oxidoreductase), *bphB* (cis-dihydrodiol dehydrogenase), *bphC* (biphenyl-2,3-diol 1,2-dioxygenase). An extra chromosomal *bphC* copy and *dmpG* (4-hydroxy-2-oxovalerate aldolase) were also detected in TG-1; *dmpG* mediates the downstream branch pathway to convert ring-cleavage intermediates into central TCA cycle carbon metabolites.

As illustrated in Figure 2, the degradation process is initiated by the stepwise hydroxylation of biphenyl to form 2,3-dihydroxybiphenyl, which is catalyzed by ring-hydroxylating dioxygenases. Subsequently, 2,3-dihydroxybiphenyl undergoes intradiol ring cleavage catalyzed by 2,3-dihydroxybiphenyl dioxygenase, generating the key ring-opening intermediate HOPDA. HOPDA is further hydrolyzed and converted into small-molecule intermediates including 4-hydroxy-2-oxopentanoate, pyruvate, and acetyl-CoA. These central carbon metabolites ultimately enter the TCA cycle.

This clear and complete catabolic pathway demonstrates the robust aromatic degradation capacity of *Rhodococcus* sp. TG-1 and provides a solid mechanistic basis for its application in biphenyl-contaminated environment remediation.

### 3.3. Comparative Transcriptomic Analysis of Rhodococcus sp. With and Without Biphenyl Exposure

To reveal the global transcriptional regulatory mechanism of *Rhodococcus* sp. TG-1 under 300 mg/L biphenyl stress, comparative transcriptomic analysis was performed between the biphenyl-treated group (BP300) and the control group (BP0).

**Figure 3:**
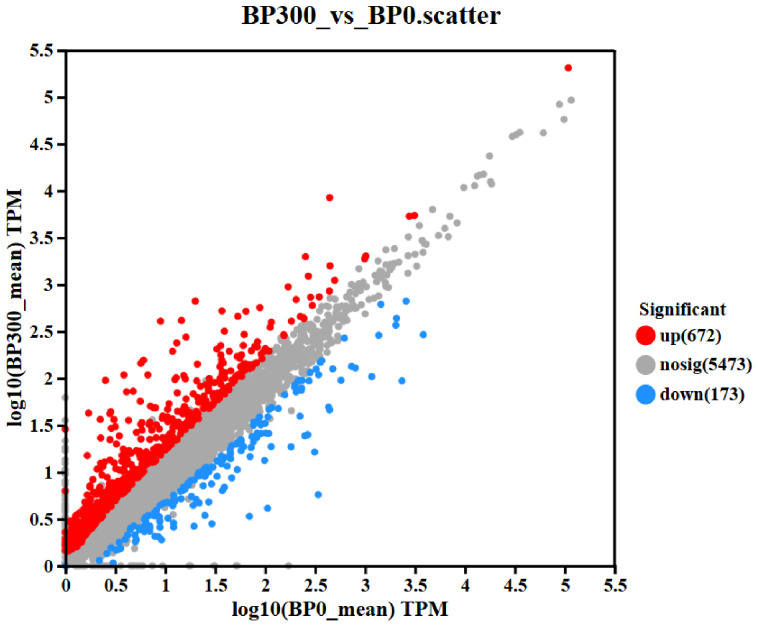
Volcano plot of differentially expressed genes (DEGs) between BP300 and BP0 groups. The x-axis represents the gene expression level in the control group (BP0) and the y-axis represents that in the biphenyl-treated group (BP300), both shown as log_10_(TPM). |log_2_FC| ≥ 1 and *p* < 0.05, 672 genes. FC: fold change; TPM: transcripts per million.

Figure 3 shows a volcano plot of differentially expressed genes (DEGs) between the two groups. A total of 845 DEGs were identified: 672 genes were significantly upregulated (red dots) and 173 were significantly downregulated (blue dots) in the BP300 group compared to the BP0 group, while the remaining 5473 genes (gray dots) showed no significant changes. The upregulated genes occupy the widest area and show the highest density, indicating that biphenyl stress primarily induces gene upregulation in this strain. Most points cluster tightly near the diagonal line, suggesting that the majority of genes maintain stable expression under biphenyl stress, and the strain’s basic metabolism and survival mechanisms remain relatively robust.

To further understand how biphenyl regulates gene expression in *Rhodococcus* sp., we performed GO and KEGG enrichment analyses on the DEGs between the BP300 and BP0 groups.

#### 3.3.1. GO Enrichment Analysis of Differentially Expressed Genes

**Figure 4:**
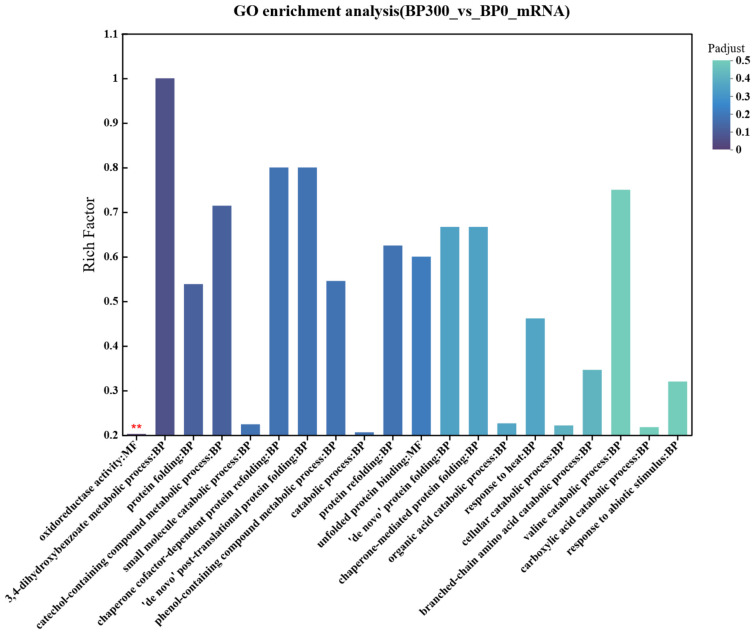
GO enrichment analysis of differentially expressed genes under biphenyl stress (BP300 vs. BP0). Note: The x-axis represents GO terms, and the y-axis represents enrichment scores. A higher value indicates a greater degree of enrichment. Colors indicate the significance of enrichment; by default, the redder the color, the more significantly enriched the GO term is. Terms with an FDR < 0.01 are marked with **.

As depicted in Figure 4, GO enrichment analysis revealed that the DEGs were significantly enriched in Molecular Function (MF) and Biological Process (BP) terms closely associated with aromatic catabolism and stress response. In the Molecular Function category, oxidoreductase activity (GO:0016491) was the most significantly enriched pathway (Padjust = 0.0028), covering 116 differentially expressed genes. These enzymes encode key catalytic proteins responsible for hydroxylation, dehydrogenation, and oxidative ring cleavage of biphenyl and its metabolic intermediates, and thus play a decisive role in the complete mineralization of biphenyl [41,42].

In the Biological Process category, “3,4-dihydroxybenzoate metabolic process” and “catechol-containing compound metabolic process” exhibited extremely high significance and rich factor values. Both 3,4-dihydroxybenzoate and catechol are critical downstream intermediates during aromatic ring cleavage and mineralization. Their significant enrichment directly verifies that strain TG-1 activates a complete aromatic catabolic pathway to utilize biphenyl as a carbon and energy source [43,44].

Meanwhile, multiple terms related to protein homeostasis were markedly enriched, including protein folding, chaperone cofactor-dependent protein refolding, and de novo post-translational protein folding. This indicates that biphenyl and its intermediates induce oxidative stress and cytotoxicity, leading to protein denaturation and misfolding. To maintain cellular function, TG-1 significantly upregulates heat shock proteins and molecular chaperones to repair damaged proteins, reflecting a robust stress-protection mechanism [45].

In addition, a series of catabolic pathways were significantly activated, including small molecule catabolism, organic acid catabolism, and carboxylic acid catabolism. These pathways are responsible for further decomposing the acidic intermediates produced during biphenyl ring cleavage to provide carbon skeletons and energy. Furthermore, the upregulation of branched-chain amino acid and valine catabolism indicates that TG-1 enhances intracellular nutrient recycling to support energy supply for detoxification and stress resistance under biphenyl exposure [44,46].

Collectively, under 300 mg/L biphenyl stress, strain TG-1 initiates a coordinated adaptive strategy: it activates the complete aromatic catabolic pathway for biphenyl degradation and utilization, while simultaneously launching a global stress response involving protein repair, oxidative stress resistance, and metabolic rearrangement. This comprehensive regulatory network ensures the survival and functional stability of TG-1 under aromatic pollutant stress.

#### 3.3.2. KEGG Enrichment Analysis of Differentially Expressed Genes

**Figure 5:**
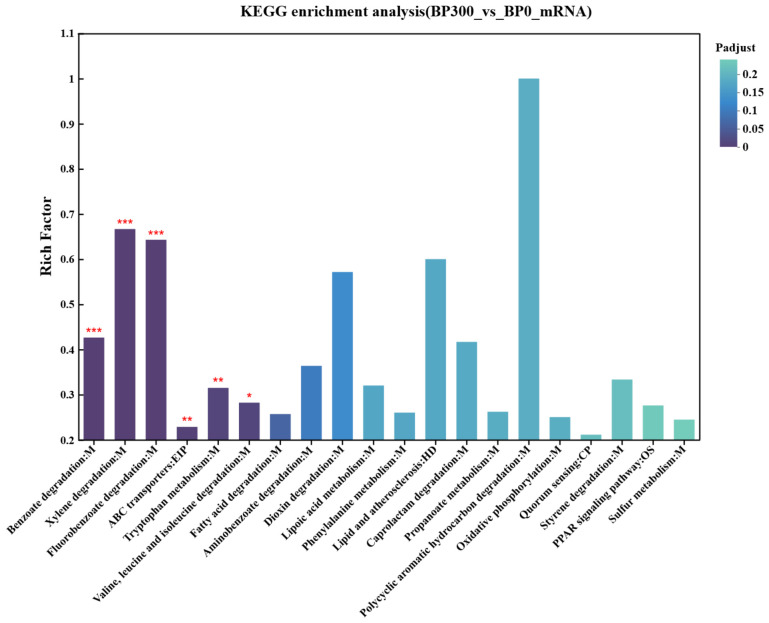
KEGG pathway enrichment analysis of differentially expressed genes under biphenyl stress (BP300 vs. BP0). Note: The x-axis represents the pathway name, and the y-axis represents the enrichment score. A higher rich factor indicates a greater degree of enrichment. The color indicates the significance of the enrichment (i.e., the *p*-value); the darker the color, the more significantly the pathway is enriched. *p*-values < 0.001 are marked with ***, *p*-values < 0.01 with **, and *p*-values < 0.05 with *; the color gradient on the right indicates the magnitude of the *p*-value.

As illustrated in Figure 5, KEGG enrichment analysis identified the core metabolic pathways activated under biphenyl stress. Benzoate degradation was the most significantly enriched pathway, which dominates the downstream ring-opening and mineralization of biphenyl metabolites [38,47]. In addition, xylene degradation, polycyclic aromatic hydrocarbon (PAH) degradation, and styrene degradation pathways were also highly activated [34]. These results confirm that strain TG-1 employs a synergistic multi-pathway network to achieve efficient and thorough catabolism of biphenyl [48].

**Figure 6:**
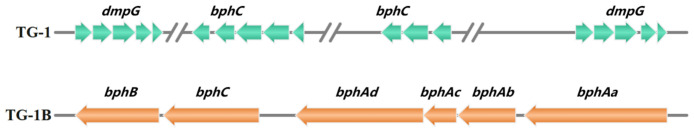
Gene clusters associated with biphenyl degradation in the genome of strain TG-1. Top track (TG-1): scattered chromosomal fragments containing two copies of *bphC* and two *dmpG* copies; double slashes represent omitted long intergenic regions. Bottom track (TG-1B): intact canonical *bph* operon composed of *bphAa*, *bphAb*, *bphAc*, *bphAd*, *bphC* and *bphB*. Arrows denote gene orientation.

Analysis of differentially upregulated genes in *Rhodococcus* sp. TG-1 successfully identified a complete gene cluster responsible for biphenyl degradation (Figure 6). Plasmid B carries a full set of biphenyl catabolic genes, including the core upper-pathway genes: *bphB* (encoding cis-dihydrodiol dehydrogenase), *bphC* (encoding biphenyl-2,3-diol 1,2-dioxygenase), *bphAd* (encoding oxidoreductase), *bphAc* (encoding the ferredoxin component of the biphenyl dioxygenase system), and *bphAa/bphAb* (encoding the large and small subunits of biphenyl 2,3-dioxygenase). Notably, two additional biphenyl degradation-related genes were identified elsewhere on the TG-1 chromosome: a second copy of *bphC* and *dmpG* (encoding 4-hydroxy-2-oxovalerate aldolase). *dmpG* functions in the lower biphenyl degradation pathway, funneling metabolites into central carbon metabolism.

#### 3.3.3. Validation of Differential Gene Expression via RT-qPCR

**Figure 7:**
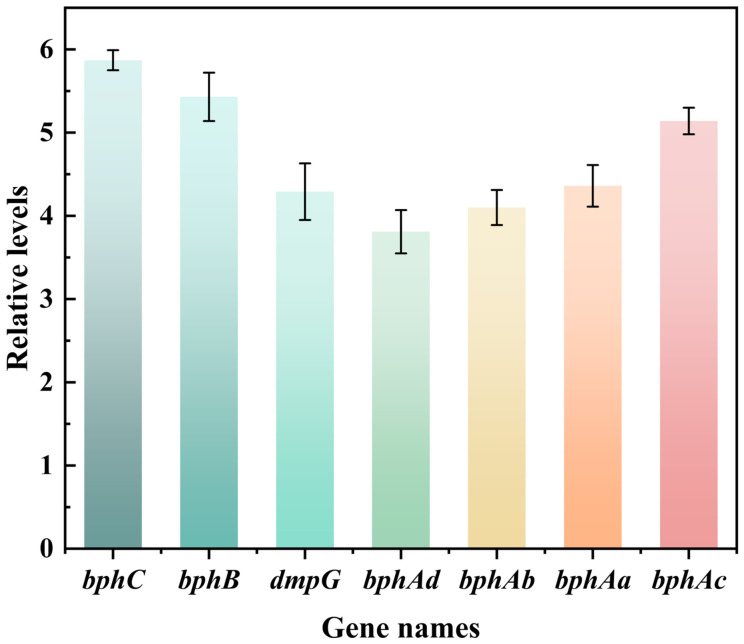
Relative expression levels of key biphenyl-degradation genes in *Rhodococcus* sp. TG-1 under 300 mg/L biphenyl stress. Error bars represent standard deviations of three biological replicates.

To validate the transcriptomic results, we performed quantitative real-time PCR (qRT-PCR) to detect the expression levels of differentially expressed genes in *Rhodococcus* sp. TG-1 under biphenyl stress. Seven key genes involved in the biphenyl degradation pathway were selected, including the upper pathway genes *bphAa/bphAb* (encoding biphenyl 2,3-dioxygenase), *bphB* (encoding cis-dihydrodiol dehydrogenase), *bphC* (encoding biphenyl-2,3-diol 1,2-dioxygenase), *bphAd* (encoding oxidoreductase), *bphAc* (encoding ferredoxin), as well as the lower pathway gene *dmpG* (encoding 4-hydroxy-2-oxovalerate aldolase). As shown in Figure 7, consistent with the RNA-seq data, the relative transcript abundances of these genes were significantly changed under biphenyl stress, highlighting their potential roles in the microbial response to biphenyl stress and biphenyl degradation.

### 3.4. Heavy Metal Tolerance Profiles of Rhodococcus sp. TG-1

An assessment was conducted of the tolerance of *Rhodococcus* sp. TG-1 to five environmentally prevalent metal ions—Cr(VI), Cd(II), Pb(II), Zn(II), and Ni(II)—at concentrations of 0–500 mg/L (Table S1). The minimum inhibitory concentration (MIC) was defined as the lowest metal concentration at which OD_600_ was below 0.4 after 72 h of incubation. Cr(VI) and Ni(II) exhibited the most pronounced inhibitory effects, completely suppressing growth at 500 mg/L, giving an MIC of 500 mg/L for both. In contrast, TG-1 demonstrated growth at the highest tested concentrations of Cd(II), Pb(II), and Zn(II), indicating a significantly higher level of resistance to these metals (MIC > 500 mg/L).

The presence of Cr(VI) resulted in a marked inhibition of growth, exhibiting a clear dose-dependent response [13,49]. At concentrations of ≤50 mg/L, cell density (OD600) remained comparable to the control (>1.2, ++++). At a concentration of 150 mg/L, growth was moderately reduced (1.0–1.2, +++). At concentrations ranging from 250 to 300 milligrams per litre, growth was found to be severely constrained (0.4–0.7, +). Complete inhibition occurred at 500 mg/L (0.4 µM) [16,50]. Given the high toxicity of Cr(VI) to TG-1 and its frequent coexistence with biphenyl in contaminated environments, it was selected as the model heavy metal for subsequent experiments [17,51].

**Figure 8:**
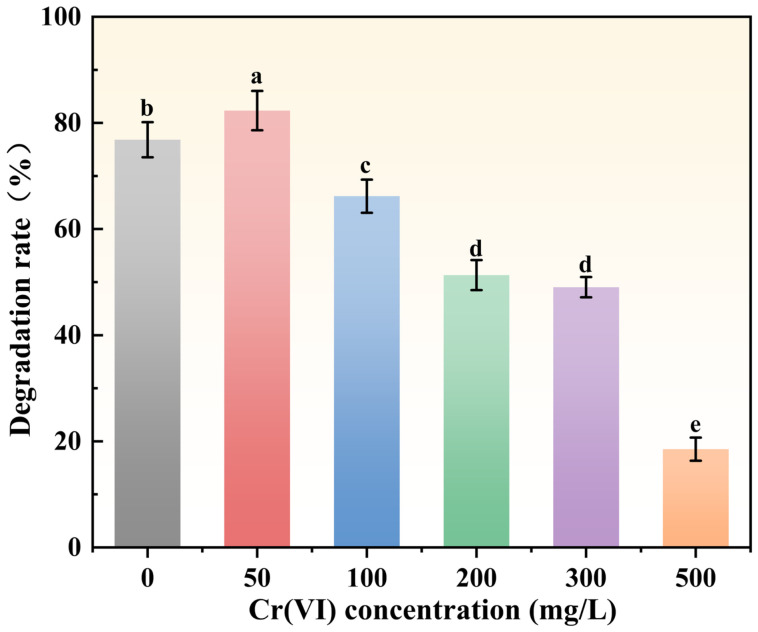
Effect of Cr(VI) on the degradation of biphenyl. Different lowercase letters above bars denote significant differences (*p* < 0.05, one-way ANOVA with Duncan’s test). Error bars show standard deviations of three biological replicates.

As demonstrated in Figure 8, the presence of Cr(VI) has a significant impact on the degradation process of biphenyl. At a concentration of 50 mg/L of Cr(VI), there was a slight yet significant increase in degradation efficiency, rising from 76.84% (absence of Cr(VI)) to 82.32%. This observation is somewhat consistent with earlier reports on the effects of low-dose heavy metals on microbial metabolic activity [52,53]. It has been hypothesised that trace Cr(VI) may act as a signalling stimulus, with the potential to activate stress-related pathways. This, in turn, may result in an indirect enhancement of the expression or activity of biphenyl-degrading enzymes [54,55].

As the concentration of Cr(VI) increased beyond 50 mg/L, a decline in degradation efficiency was observed. The percentage of the substance present in the sample at 100 mg/L was found to be 66.20%, at 200 mg/L it was 51.33%, at 300 mg/L it was 49.05%, and at 500 mg/L it was only 18.52%. This decline is indicative of the increasing toxicity of Cr(VI) [56]. At elevated concentrations, Cr(VI) can induce severe oxidative stress, disrupt cell membrane integrity, inhibit key metabolic enzymes, and impair normal physiological functions, ultimately suppressing biphenyl degradation [55,57]. Although 500 mg/L Cr(VI) completely inhibited cell growth (MIC), a residual degradation efficiency of 18.52% was still observed after 3 days, which may be attributed to extracellular enzyme activity or partial degradation before complete growth inhibition [58].

As 200 mg/L Cr(VI) significantly inhibited but did not completely halt biphenyl degradation (51.33%), this concentration was selected for subsequent dual-contaminant degradation experiments [53,59]. This condition provides a quantifiable level of toxic stress while retaining sufficient degradation activity to evaluate the biochar-based system. In summary, TG-1 displays robust biphenyl-degrading activity under moderate Cr(VI) stress conditions, thereby underscoring its potential for the degradation of sites contaminated with biphenyl and Cr(VI) in a co-contaminated state [58].

### 3.5. Modification and Characterization of Straw Biochar

**Figure 9:**
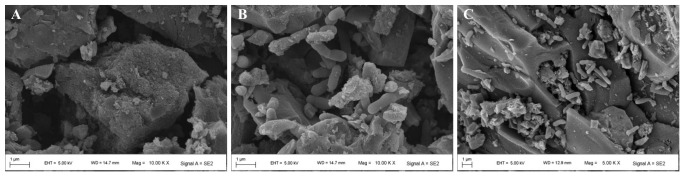

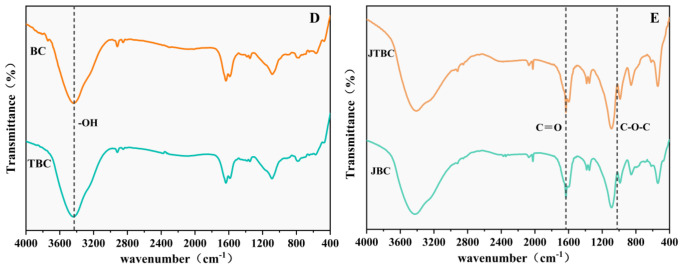
TG−1 immobilized on biochar: (**A**) Scanning electron microscope (SEM) image of straw biochar; (**B**) Scanning electron microscope (SEM) image of TG−1 immobilized on straw biochar; (**C**) Scanning electron microscopy (SEM) image of TG−1 immobilized on alkali-modified straw biochar; (**D**) Fourier transform infrared (FTIR) spectra of TG−1 before and after adsorption onto straw biochar (BC refers to raw straw biochar, and TBC refers to straw biochar adsorbed with TG−1); (**E**) Fourier transform infrared (FTIR) spectra of TG−1 before and after adsorption onto alkali-modified straw biochar (JBC stands for alkali-modified straw biochar, and JTBC stands for alkali-modified straw biochar adsorbed with TG−1).

SEM was employed to characterize the microstructure of biochar and the immobilization of TG-1 (Figure 9). Raw straw biochar exhibited a loose and smooth surface (Figure 9A). After immobilization, TG-1 cells were clearly adsorbed on the surface (Figure 9B). In previous laboratory studies, SEM images of alkali-modified straw biochar revealed a porous structure with flake-like fragments and a noticeably rougher surface, which should favor bacterial attachment. FTIR analysis indicated that the peaks corresponding to oxygen-containing functional groups, such as hydroxyl (–OH) and carboxyl (–COOH), became much stronger after alkali modification, enhancing interactions with heavy metals and microbial cells. XPS further confirmed the enrichment of oxygen-containing groups on the biochar surface, which can complex with metal ions and improve adsorption capacity. BET analysis showed that the specific surface area of alkali-modified straw biochar increased from 56.27 m^2^/g to 109.30 m^2^/g, with an average pore size of 5.96 nm, providing more active sites for pollutant adsorption and microbial colonization [31,32]. After loading with strain TG-1, a greater number of bacterial cells were attached and uniformly distributed, indicating that this modified biochar possesses a stronger capacity for microbial immobilization (Figure 9C).

FTIR was used to analyze surface functional groups before and after immobilization (Figure 9D). Compared with raw BC, TBC exhibited a stronger –OH absorption peak near 3400 cm^−1^, indicating that TG-1 binds to the biochar surface mainly through hydrogen bonding and electrostatic interactions involving hydroxyl and carboxyl groups.

Figure 9E compares alkali-modified biochar (JBC) and TG-1-loaded alkali-modified biochar (JTBC). JBC showed stronger carboxyl (–COOH) and ether (C–O–C) peaks at 1600 cm^−1^ and 1050 cm^−1^, confirming that alkali modification introduced more active functional groups. The spectral shifts were more pronounced between JTBC and JBC than between TBC and BC, suggesting that alkali-modified biochar, with its richer surface functional groups, immobilizes TG-1 more effectively and provides a more stable carrier for degradation.

### 3.6. Removal of Cr(VI) and Biphenyl in a Dual-Contaminant System by the Immobilized Strain TG-1

To alleviate Cr(VI)-induced cytotoxicity and enhance the biphenyl removal efficiency of TG-1 under combined pollution stress, an immobilized bioremediation system (JTBC) was constructed using alkali-modified straw biochar as the carrier. Uninoculated alkali-modified biochar (JBC) was set as the abiotic control group.

**Figure 10:**
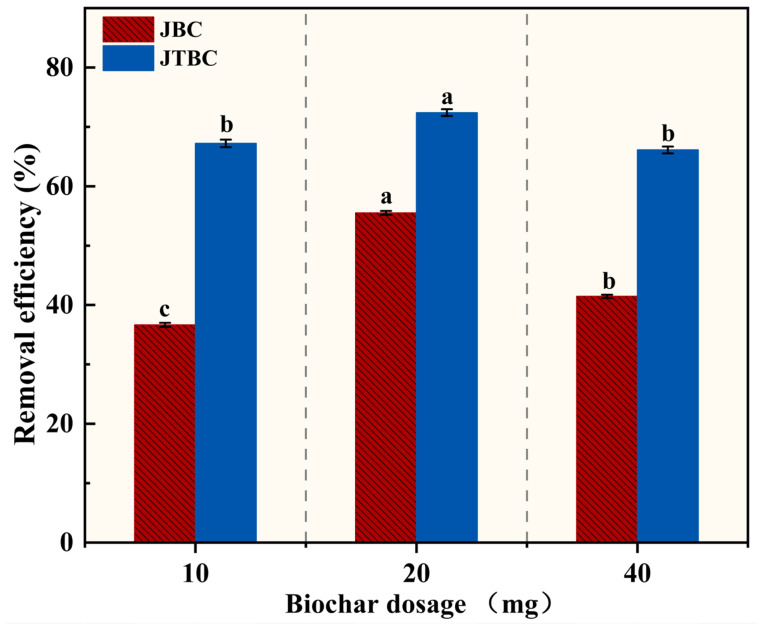
Effect of biochar dosage on biphenyl removal. JBC (abiotic control) represents adsorption removal by alkali-modified biochar alone; JTBC (immobilized system) represents total removal by biochar-immobilized TG-1. Different lowercase letters above each bar indicate significant differences between treatments (one-way ANOVA, Duncan’s multiple range test, *p* < 0.05). Data are shown as mean ± SD (*n* = 3).

The effect of biochar dosage on biphenyl removal is presented in Figure 10. For abiotic JBC, the maximum adsorption removal rate of 55.40% was achieved at 20 mg, with lower values of 36.60% (10 mg) and 41.40% (40 mg), which was attributed to the limited adsorption capacity of biochar alone. For the immobilized system JTBC, the removal efficiency increased from 67.40% (10 mg) to 72.40% (20 mg), but decreased to 66.40% at 40 mg. Under the optimal dosage of 20 mg, the biphenyl removal rate achieved by JBC through physical adsorption was 55.40%, while the total removal rate of JTBC reached 72.40%. JTBC exhibited higher removal efficiency compared to JBC, which is attributed to its combined effects of adsorption and the activity of immobilized microorganisms, jointly promoting biphenyl removal. The reduced efficiency at high dosage can be explained by the over-adsorption of biphenyl on biochar, which reduces bioavailability for microbial removal [23,59]; meanwhile, excessive carriers tend to agglomerate, hindering mass transfer of nutrients and oxygen and causing local substrate competition [24,59].

At all dosages, the removal efficiency of JTBC was higher than that of JBC, suggesting that the degradation activity of the immobilized microorganisms provided an additional removal capacity beyond adsorption. However, physical adsorption remained the major contributor to the overall removal. Therefore, 20 mg was determined as the optimal biochar dosage for subsequent combined pollution degradation experiments.

**Figure 11:**
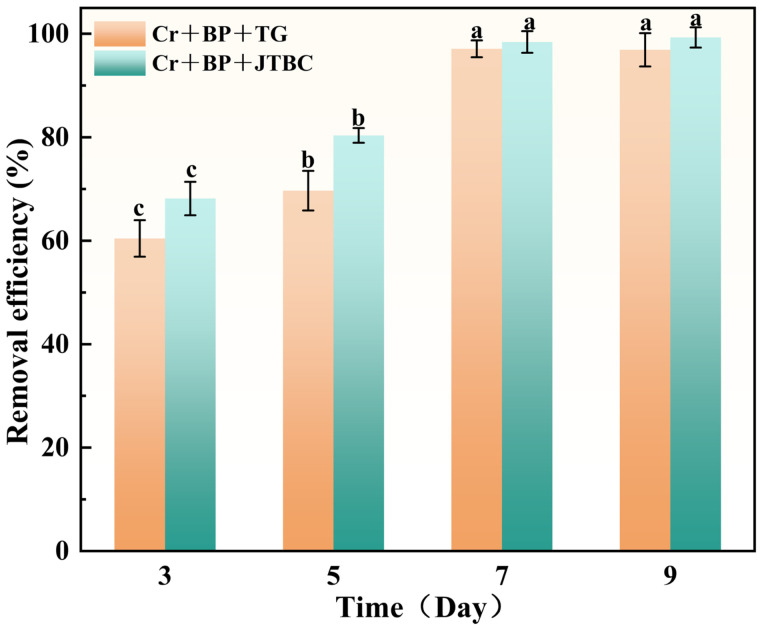
Removal rate of biphenyl by immobilized TG-1 under biphenyl-Cr(VI) co-contamination conditions. Different lowercase letters above bars represent significant intergroup differences (one-way ANOVA, Duncan’s test, *p* < 0.05). Data are mean ± SD of three replicates.

Figure 11 compares biphenyl degradation by free TG-1 (Cr + BP + TG) and immobilized JTBC (Cr + BP + JTBC) during 9 days of incubation under 200 mg/L Cr(VI) stress. Both groups showed time-dependent increasing degradation, and the immobilized system consistently exhibited superior performance. On day 3, JTBC achieved 68.15% degradation, significantly higher than the 60.44% of free cells. On day 5, the degradation efficiency reached 80.37% for JTBC and 69.67% for free TG-1. By day 7, both groups achieved nearly complete degradation: 97.10% for free TG-1 and 98.42% for JTBC. On day 9, the final removal efficiencies were 96.92% and 99.30%, respectively. At the optimal biochar dosage of 20 mg (as determined in Figure 9), abiotic JBC alone removed 55.40% of biphenyl through physical adsorption.

Notably, free TG-1 exhibited much higher degradation efficiency in the co-contamination system (60.44% on day 3; 97.10% on day 7) than in the single Cr(VI) stress system (51.33%, Section 3.4). This improvement can be attributed to the fact that biphenyl acts as a carbon and energy source to support cell growth and partially alleviate Cr(VI) toxicity [60].

Despite the enhanced performance of free cells, the immobilized system still achieved significantly higher degradation rates, confirming that biochar immobilization provides effective protection against Cr(VI) toxicity [59]. The biochar matrix acts as a physical barrier to reduce direct contact between microbial cells and Cr(VI) [23]; meanwhile, its abundant surface functional groups adsorb Cr(VI) and alleviate oxidative stress, thus maintaining high metabolic activity and biphenyl degradation capacity [13,59].

These results demonstrate that alkali-modified biochar immobilization significantly enhances the heavy metal tolerance and catalytic stability of *Rhodococcus* sp. TG-1, enabling efficient biphenyl degradation under Cr(VI) co-contamination. The JTBC system therefore shows high potential for the treatment of sites co-contaminated with biphenyl and Cr(VI), focusing on the removal of the organic pollutant in the presence of the heavy metal.

## 4. Conclusions

This study investigated the biphenyl-degrading strain *Rhodococcus sp.* TG-1, focusing on its degradation conditions, metabolic pathways, transcriptomic responses, Cr(VI) tolerance, and immobilized degradation performance. The optimal temperature and pH for biphenyl degradation were 30 °C and 7.0, with stable activity at 100–900 mg/L biphenyl. LC-MS analysis confirmed that TG-1 degrades biphenyl via the 2,3-dihydroxybiphenyl dioxygenase pathway, generating intermediates such as HOPDA, which are further decomposed and enter the tricarboxylic acid cycle.

Through transcriptomic analysis, this study provided deeper molecular insights. Under biphenyl stress, 845 differentially expressed genes and seven complete *bph* gene clusters were identified, revealing the genetic basis of biphenyl catabolism in this strain. TG-1 showed high Cr(VI) tolerance (MIC 500 mg/L) and maintained efficient degradation under low-to-moderate Cr(VI) stress. SEM and FTIR confirmed stable immobilization of TG-1 on raw and alkali-modified straw biochar, and the immobilized agent outperformed free bacteria in co-contaminated systems. In addition, the application of alkali-modified straw biochar as an immobilization carrier adds a practical and environmentally friendly dimension to the study. Taken together, the integration of degradation kinetics, pathway analysis, transcriptomics, and biochar immobilization makes this work relatively comprehensive in addressing biphenyl degradation under heavy metal stress. Overall, TG-1 immobilized on alkali-modified straw biochar (JTBC) offers an efficient and green strategy for enhancing biphenyl removal in the presence of Cr(VI), showing promise for the treatment of co-contaminated sites.

## Data Availability

All experimental data supporting our findings are stored in our laboratory, and relevant raw data can be obtained from the corresponding author upon reasonable request. No public archived datasets were generated in this research.

## Supplementary Materials

The following supporting information can be downloaded at: https://www.mdpi.com/article/10.3390/microorganisms14061384/s1, Table S1: Heavy metal tolerance profiles of *Rhodococcus* sp. TG-1 against five heavy metals; Figure S1: HPLC chromatograms of biphenyl degradation by *Rhodococcus* sp. TG-1. A, Biphenyl; B, 2,3-Dihydroxybiphenyl; C, 2-Hydroxy-6-oxo-6-phenylhexa-2,4-dienoic acid (HOPDA); D, 2-Hydroxypenta-2,4-dienoic acid; E, 2-Oxoglutaric acid (α-Ketoglutaric acid).
