# Supplementary material for: Enhanced Biphenyl Degradation by Rhodococcus sp. TG-1 Under Cr(VI) Stress via Modified Biochar Immobilization
Source: Microorganisms. 2026 Jun 22;14(6):1384. doi: 10.3390/microorganisms14061384 (PMC13304454; doi:10.3390/microorganisms14061384)
Supplement: Supplementary file 1 [file microorganisms-14-01384-s001.zip › microorganisms-4358278-supplementary.pdf]

[Supplementary information]

Enhancement of the Remediation Efficiency of *Rhodococcus* sp. TG-1 for Biphenyl and Cr(VI) by Modified Biochar

Supplementary Sections

List of Tables

Table S1 Heavy metal tolerance profiles of *Rhodococcus* sp. TG-1 against five heavy metals.

Metal Ions	Metal Ion Concentration (mg/L)						MIC (mg/L)
	0	50	150	250	300	500	
**Cr(VI)**	++++	++++	+++	+	+	-	500
**Cd(II)**	++++	++	+	+	+	+	＞500
**Pb(II)**	++++	++++	+++	+++	+++	++	＞500
**Zn(II)**	++++	++++	++++	+++	++	++	＞500
**Ni(II)**	++++	++++	+	+	+	-	500

Note: - : OD600 < 0.4; + : 0.4 < OD600 < 0.7; ++: 0.7 < OD600 < 1; +++ : 1 < OD600 < 1.2; ++++ : OD600 > 1.2.

Figure S1 HPLC chromatograms of biphenyl degradation by *Rhodococcus* sp. TG-1. A、Biphenyl; B、2,3-Dihydroxybiphenyl; C、2-Hydroxy-6-oxo-6-phenylhexa-2,4-dienoic acid (HOPDA); D、2-Hydroxypenta-2,4-dienoic acid; E、2-Oxoglutaric acid (α-Ketoglutaric acid).

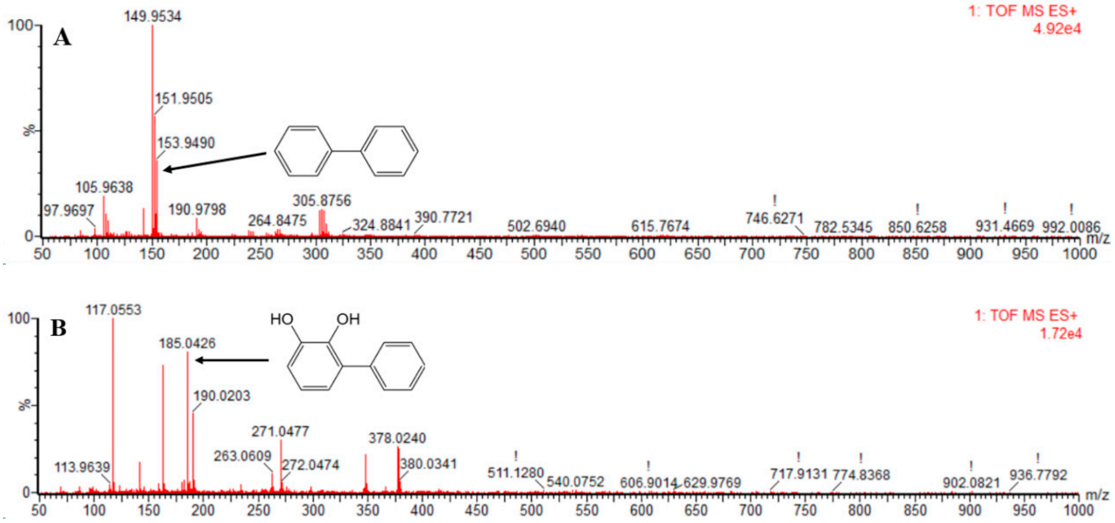

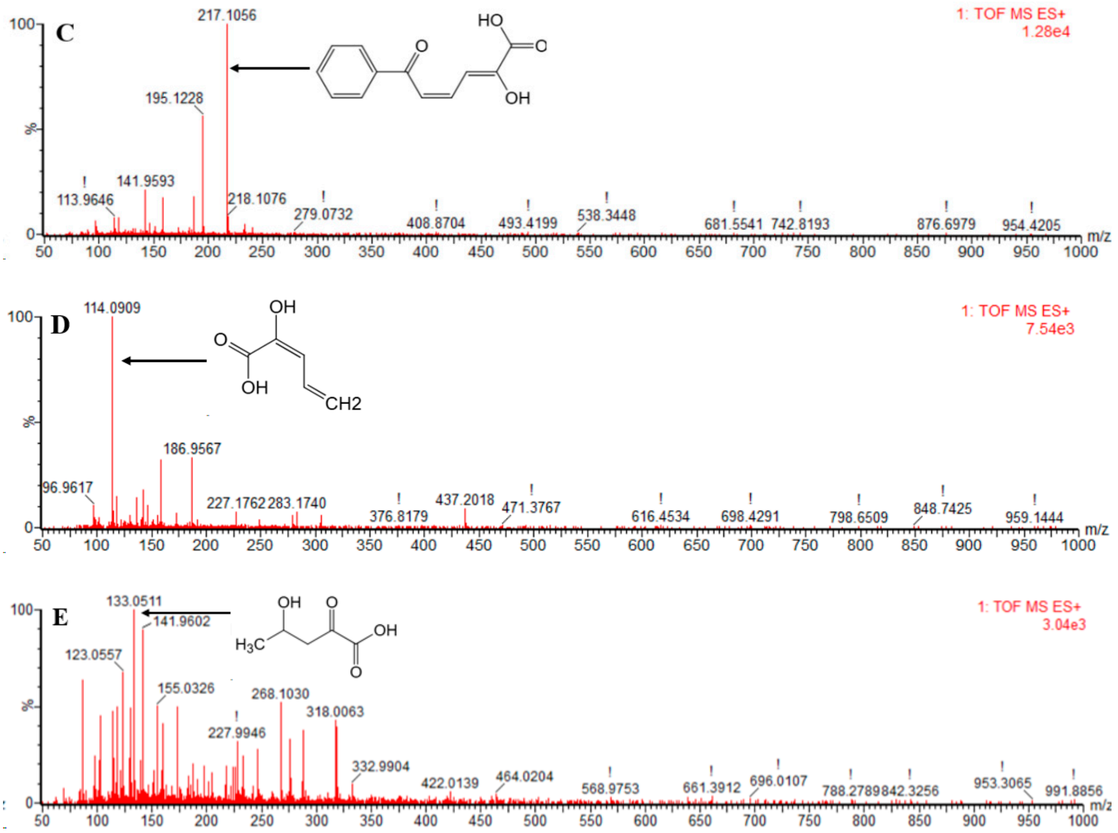
C
217.1056
195.1228
141.9593
113.9646
218.1076
279.0732
408.8704
493.4199
538.3448
681.5541
742.8193
876.6979
954.4205
1: TOF MS ES+
1.28e4
HO
OH
D
114.0909
186.9567
96.9617
227.1762
283.1740
376.8179
437.2018
471.3767
616.4534
698.4291
798.6509
848.7425
959.1444
1: TOF MS ES+
7.54e3
OH
CH2
E
133.0511
141.9602
123.0557
155.0326
268.1030
318.0063
227.9946
332.9904
422.0139
464.0204
568.9753
661.3912
696.0107
788.2789
842.3256
953.3065
991.8856
1: TOF MS ES+
3.04e3
H_3C
%
m/z
